# Spatial and temporal trend analysis of the burden of endocrine-related cancers among women of reproductive age in the Asia-Pacific region from 1990 to 2021: results based on the GBD study

**DOI:** 10.3389/fonc.2025.1678501

**Published:** 2026-01-21

**Authors:** Chengchi Xia, Baoqing Wang

**Affiliations:** Department of Oncology, The Second Affiliated Hospital of Xuzhou Medical University, Jiangsu, China

**Keywords:** Asia, breast neoplasms, global burden of disease, ovarian neoplasms, reproductive age, social determinants of health, thyroid neoplasms, women’s health

## Abstract

**Background:**

Endocrine-related cancers pose an escalating challenge for reproductive-age women in the Asia-Pacific region, characterized by persistent socioeconomic disparities.

**Methods:**

Using data from the Global Burden of Disease Study 2021, we analyzed the incidence, mortality, and DALYs of breast, ovarian, and thyroid cancers across 15 countries (1990–2021). Trends were quantified using EAPC, and mortality trajectories through 2050 were projected using GAM.

**Results:**

Breast cancer exhibited a polarized pattern: mortality steadily declined in High-SDI nations but surged in Low-SDI regions. Thyroid cancer revealed a dichotomy of screening-driven overdiagnosis in High-SDI settings versus high lethality in Low-SDI areas. Ovarian cancer maintained the poorest prognosis in resource-limited settings. Crucially, primary risk drivers are shifting from traditional behavioral factors to metabolic factors.

**Conclusion:**

With disparities projected to widen by 2050, stratified interventions are urgent. We recommend screening de-escalation for High-SDI nations and resource-adapted measures for Low-to-Middle SDI nations to bridge the growing equity gap. reproductive-age women.

## 1 Introduction

Endocrine-related cancers have emerged as a major and increasingly severe public health challenge among reproductive-age women (15–49 years) worldwide (1, 2). Traditional cancer research has long focused on older populations (3), resulting in the underestimation of the epidemiological characteristics, risk factors, and intervention needs of reproductive-age women. This oversight has led to significant data gaps and research blind spots (4, 5). However, these malignancies are not only associated with high mortality and disability rates but also coincide with the most economically productive stage of a woman’s life (6–8), thereby amplifying their broader societal costs. The Asia-Pacific region, home to half of the global population, has experienced a substantial increase in the burden of endocrine-related cancers over the past three decades (9, 10).

The Asia-Pacific region accommodates nearly half of the world’s population; consequently, reproductive-age women in this region shoulder a disproportionately large fraction of the global burden of endocrine-related cancers (1). Over the past three decades, rapid urbanization, progressively delayed childbearing, escalating obesity prevalence, and heightened exposure to environmental endocrine-disrupting chemicals have collectively driven a marked increase in the incidence of these malignancies across Asia-Pacific (11–13). To quantify this evolving burden, we selected 15 representative countries within the region and conducted a comprehensive assessment of three high-incidence, clinically significant endocrine-related cancers—breast, ovarian, and thyroid cancers—among women aged 15–49 years. Leveraging data from the Global Burden of Disease Study 2021, we calculated age-standardized incidence rates, mortality rates, and disability-adjusted life-years (DALYs) for the period 1990–2021. The estimated annual percentage change (EAPC) was computed for each metric, and geographical heterogeneity was examined with the Socio-demographic Index (SDI). A validated predictive model was further employed to project disease trajectories through 2050.

By delineating epidemiological patterns, quantifying disease burden, and delineating temporal trends, this study provides an evidence-based foundation for the formulation of targeted prevention and control strategies for endocrine-related cancers in the Asia-Pacific region.

## 2 Methods

### 2.1 Data source

This study did not involve human participants, and all data were obtained from the Global Burden of Disease Study 2021 (GBD 2021), curated by the Institute for Health Metrics and Evaluation (IHME) and publicly available through the Global Health Data Exchange (GHDx) platform (https://ghdx.healthdata.org/gbd-2021). The GBD study provides a freely accessible and comprehensive database of attributable disease burden estimates for all countries and risk factors, generated using standardized and peer-reviewed methodologies. This analysis adheres to the Guidelines for Accurate and Transparent Health Estimates Reporting (GATHER), ensuring transparency and reproducibility in the use and presentation of health data.

### 2.2 Statistical analysis

Temporal trends in the Age-Standardized Rate (ASR) were first quantified using the Estimated Annual Percentage Change (EAPC), a standard metric for summarizing dynamic shifts in disease burden. A log-linear regression model was fitted to the natural logarithm of the ASR against the calendar year, described as ln(ASR) = α + β × Year + ϵ. The EAPC was calculated as 100 × (exp(β) - 1), with 95% confidence intervals (CIs) derived from the standard error of the regression coefficient (β). Trends were defined as significant if the 95% CI excluded zero: an ASR was considered to be increasing if the lower bound was greater than zero and decreasing if the upper bound was less than zero.

To further evaluate disparities in these cancer burdens across development gradients, the Socio-demographic Index (SDI) was utilized. The SDI is a composite metric developed by GBD researchers to assess the socioeconomic status of a region. It integrates per capita income, average years of schooling, and total fertility rates into a single metric ranging from 0 to 1, reflecting the socioeconomic health and progress of a region or country. Following the standard stratification framework of the GBD 2021 study, countries were categorized into five quintiles based on specific SDI cut-off values: Low (0–0.454743), Low-middle (0.454743–0.607679), Middle (0.607679–0.689504), High-middle (0.689504–0.805129), and High SDI (0.805129–1) (14). This quintile-based stratification facilitated a granular comparative analysis of disease burden and temporal trajectories across varying socioeconomic levels. Concurrently, the contributions of specific risk factors to the observed cancer burden were assessed using the Summary Exposure Value and Population Attributable Fraction (PAF) extracted from the GBD Risk Factors framework.

Regarding future projections, mortality trends through 2050 were modeled using a Generalized Additive Model (GAM) integrated with penalized smoothing splines. This non-parametric framework was specifically adopted for its superior flexibility in capturing complex, non-linear temporal fluctuations that traditional rigid parametric models (such as linear regression) may fail to capture (15, 16). The model was specified as Rate ~ s(Year), where s(Year) denotes a smoothing function allowing the data distribution to determine the trend curve’s shape. Generalized Cross-Validation (GCV) was employed to automatically select the optimal degree of smoothness, thereby minimizing the risk of overfitting. To rigorously account for stochastic uncertainty in long-term forecasting, 95% uncertainty intervals (UIs) were generated through 1,000 simulations from the Bayesian posterior distribution of the model parameters rather than relying solely on standard errors.

Additionally, to quantify the magnitude of changes in disease burden, we calculated the absolute difference in Age-Standardized Rates (ASR) between 1990 and 2021. Furthermore, the Mortality-to-Incidence Ratio (MIR), defined as the ratio of ASMR(age-standardized mortality rate) to ASIR(age-standardized incidence rate), was computed to serve as a proxy for cancer prognosis and clinical management efficacy (17). All statistical analyses and visualizations were performed using R software (version 4.5.0).

## 3 Result

### 3.1 Time series trend analysis of endocrine-related cancers in reproductive-age women (1990-2021)

**Figure 1:**
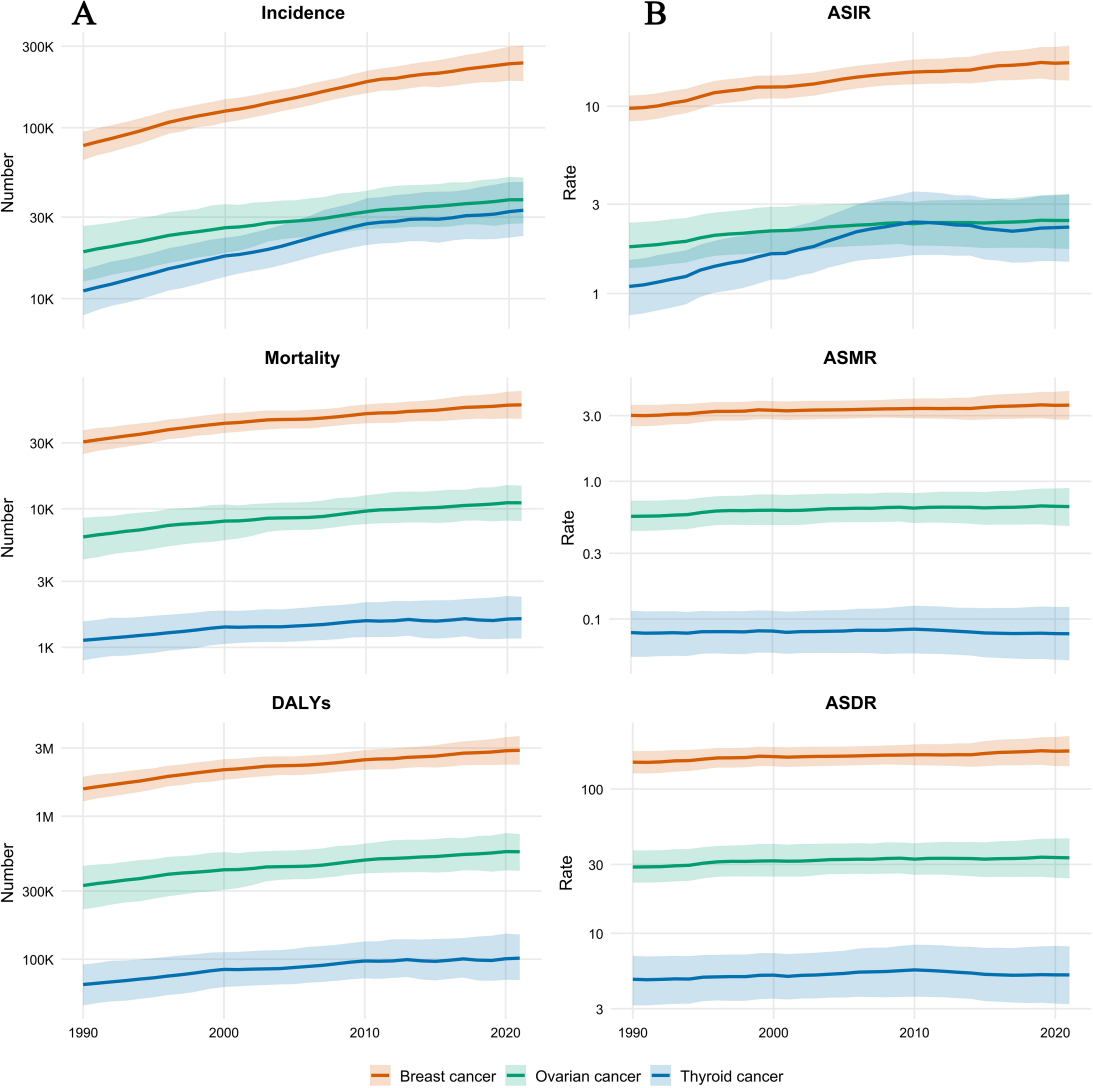
Overall temporal trends in the burden of endocrine-related cancers among reproductive-age women in the Asia-Pacific Region, 1990-2021. **(A)** Temporal trends of absolute numbers for Incidence cases, Deaths, and Disability-Adjusted Life Years (DALYs). Note that the Y-axis is presented on a log10 scale to accommodate the wide range of values across different cancer types. **(B)** Temporal trends of Age-Standardized Rates (ASR), including Age-Standardized Incidence Rate (ASIR), Age-Standardized Mortality Rate (ASMR), and Age-Standardized DALY Rate (ASDR). The solid lines represent the central estimates, while the shaded bands surrounding the trend lines indicate the 95% uncertainty intervals (UIs). Different colors denote specific cancer types: Breast cancer (Orange), Ovarian cancer (Green), and Thyroid cancer (Blue). reproductive-age women.

Between 1990 and 2021, the burden of endocrine-related cancers among reproductive-age women in the Asia-Pacific region underwent pronounced shifts. Overall, the absolute burden (incident cases, deaths, and DALYs) rose markedly, reflecting the growing demand on healthcare systems, while the epidemiological risk (ASR) showed varied trends across cancer types (**Figure 1**).

**Figure 2:**
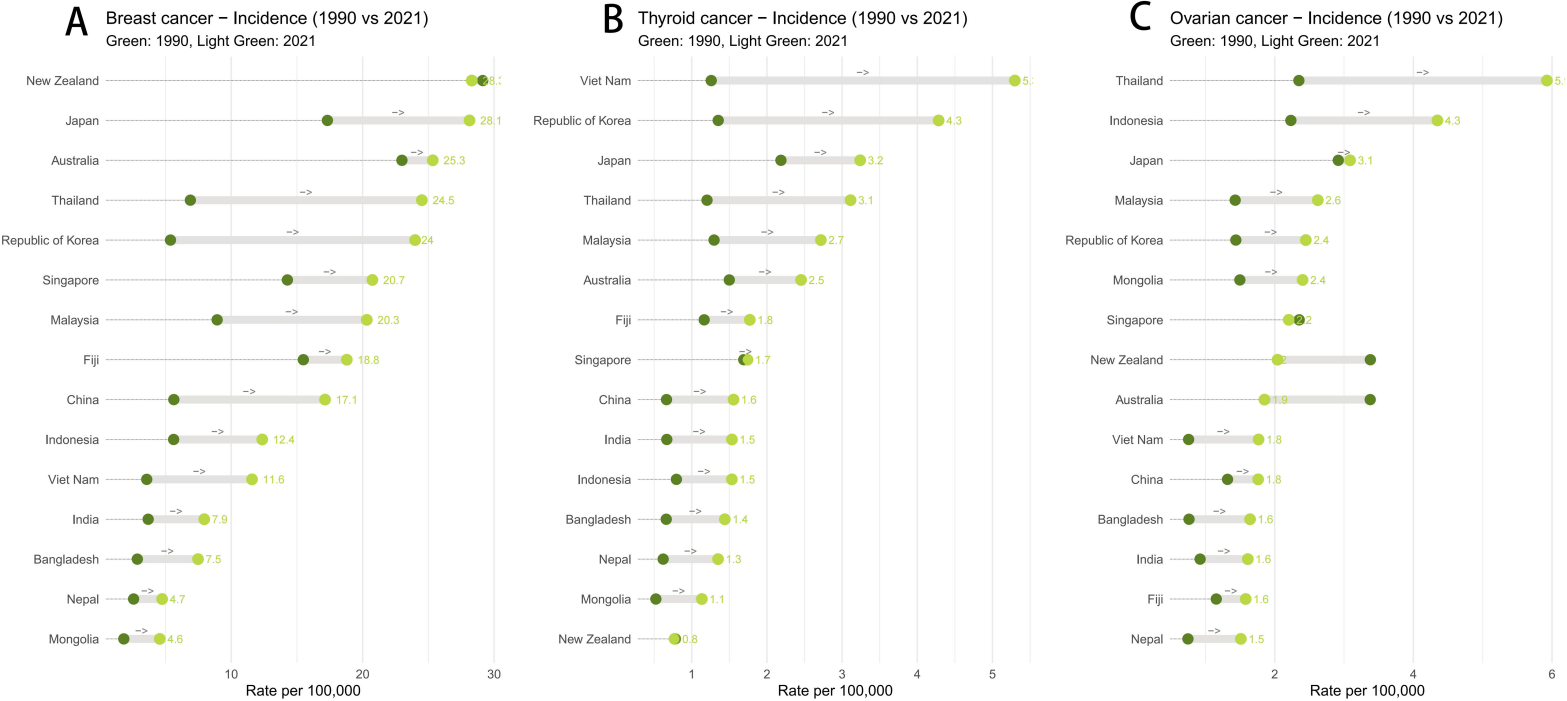
Temporal shifts in the burden of endocrine-related cancers among reproductive-age women in the Asia-Pacific region (1990–2021). **(A)** Breast cancer, **(B)** Thyroid cancer, and **(C)** Ovarian cancer. This dumbbell chart illustrates the change in ASIR over three decades. The dark green dots represent the baseline rate in 1990, while the light green dots correspond to the rate in 2021. The length of the connecting gray line signifies the magnitude of the change. A “rightward shift” indicates an increase in disease burden, whereas a “leftward shift” indicates a reduction.

To quantify the magnitude of these shifts, we analyzed the absolute changes in age-standardized rates (**Figure 2**). As illustrated in the dumbbell plot, South Korea and Vietnam exhibited the most dramatic “rightward shifts.” Specifically, breast cancer incidence in South Korea surged at an alarming rate, more than tripling to 5,619.6 cases in 2021. Similarly, Vietnam witnessed a striking expansion in thyroid cancer, with cases multiplying nearly six-fold to 2,734. Thailand also saw a continuous rise, emerging with the region’s highest ovarian cancer incidence burden (1,952 new cases), underscoring severe prevention challenges.

Mortality trends revealed a contrasting “East vs. West” pattern. Thailand faced significant increases in deaths from both breast (reaching 1,810.3) and ovarian cancers (reaching 2,388.7), suggesting difficulties in disease control. In stark contrast, Japan demonstrated a unique “leftward shift” in the dumbbell plot, achieving a reduction in absolute disease risk with declining deaths for both malignancies. Furthermore, thyroid cancer mortality showed prominent heterogeneity: while deaths steadily declined in high-SDI nations like South Korea and New Zealand, they showed an upward trend in India, rising to 928.2 deaths in 2021.

Age-stratified analyses further revealed distinct age-specific patterns in the distribution of endocrine-related cancers. In 2021, the global disease burden of endocrine-related cancers among reproductive-age women was highly concentrated in the 45–49 age group (**Supplementary Figure S1**). Across all indicators—including new cases, deaths, and DALYs—both absolute numbers and age-specific rates increased with age, peaking in this subgroup.

### 3.2 SDI-driven disparities in endocrine-related cancer burden among reproductive-aged women in the Asia-Pacific Region(1990-2021)

**Figure 3:**
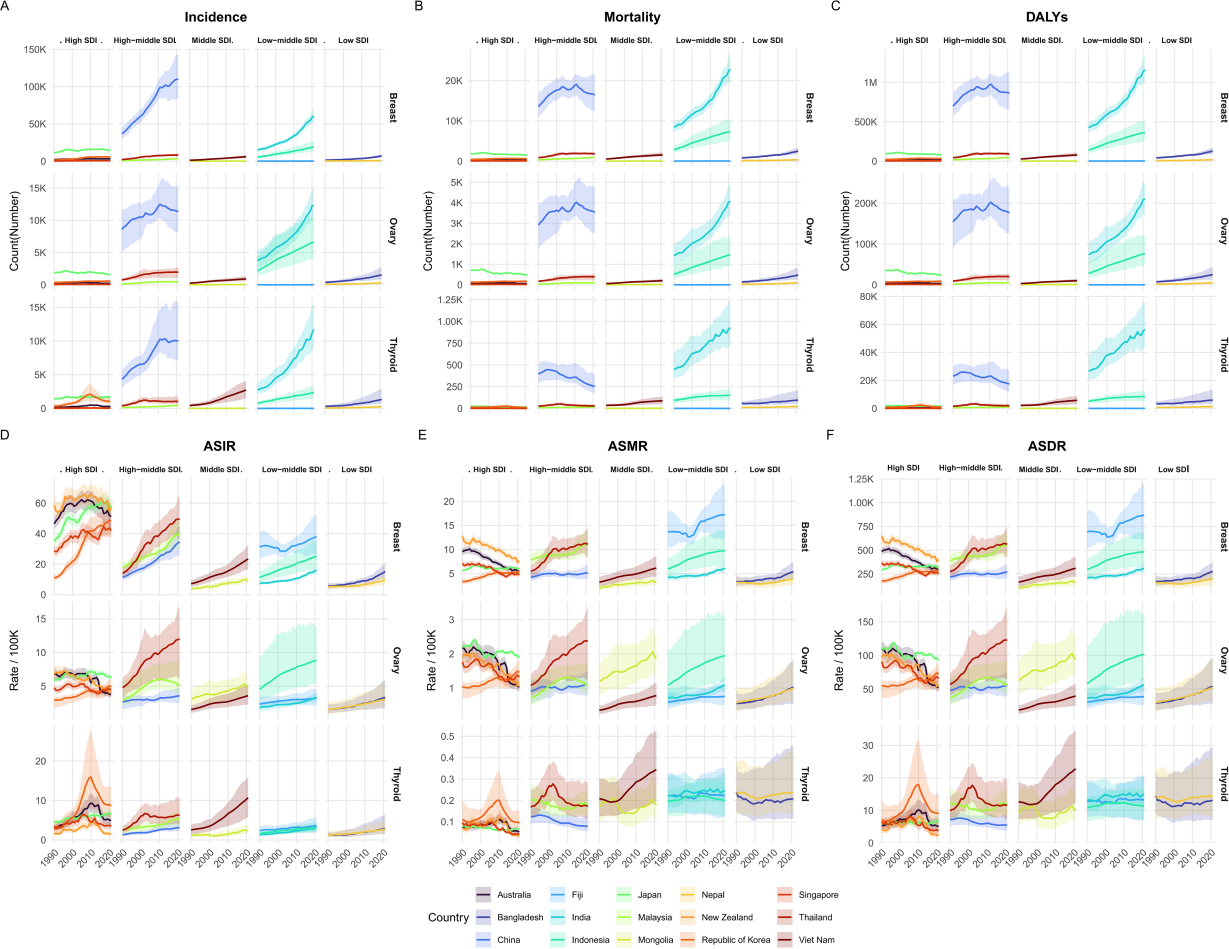
Trends in the burden of endocrine-related cancers stratified by Socio-demographic Index (SDI) quintiles (1990-2021). The upper panels display the trends in Absolute Numbers for **(A)** Incidence, **(B)** Deaths, and **(C)** DALYs. The lower panels display the trends in Age-Standardized Rates for **(D)** ASIR, **(E)** ASMR, and **(F)** ASDR. Note: Countries are categorized into five SDI quintiles (High, High-middle, Middle, Low-middle, and Low). Shaded areas indicate the 95% uncertainty intervals (UIs) associated with the estimates.

From an SDI stratification perspective, the burden of endocrine-related cancers among reproductive-age women in the Asia-Pacific region demonstrated marked disparities across SDI levels. Countries with high SDI are significantly more effective in cancer prevention and control than countries with medium to low SDI, highlighting the huge gaps in medical capacity, early detection, and resource allocation between countries at different levels of development (**Figure 3**).

In high-SDI countries, such as New Zealand and Australia, substantial declines in ASMR and age-standardized ASDR for endocrine-related cancers were achieved, despite a continued upward trend in ASIR.

For instance, in New Zealand, the ASMR for breast cancer decreased from 6.3 per 100,000 (95% CI: 5.8–6.8) in 1990 to 3.8 per 100,000 (95% CI: 3.5–4.1) in 2021, with an EAPC of –1.49 (95% CI: –1.66 to –1.32). Similarly, the ASMR for ovarian cancer declined from 0.9 per 100,000 (95% CI: 0.8–1.1) to 0.5 per 100,000 (95% CI: 0.5–0.6), with an EAPC of –1.89 (95% CI: –2.12 to –1.66). In Australia, the ASMR for breast cancer fell from 4.7 per 100,000 (95% CI: 4.4–5.1) to 2.7 per 100,000 (95% CI: 2.4–3.0), with an EAPC of –2.16 (95% CI: –2.30 to –2.03), while the ASMR for ovarian cancer decreased from 1.1 per 100,000 (95% CI: 0.9–1.2) to 0.5 per 100,000 (95% CI: 0.5–0.6), with an EAPC of –2.35 (95% CI: –2.81 to –1.88).

These consistent reductions in mortality and disease burden highlight the effectiveness of early detection, improved treatment strategies, and robust healthcare infrastructure in high-SDI settings. By contrast, although the overall mortality rate of thyroid cancer in the Asia-Pacific region has remained relatively stable, its incidence has increased markedly across both high- and low-SDI countries. In South Korea, the ASIR rose from 1.4 per 100,000 (95% CI: 0.9–2.4) in 1990 to 4.3 per 100,000 (95% CI: 2.7–6.7) in 2021, with an EAPC of 4.93 (95% CI: 3.58–6.30). In China, the ASIR increased from 0.7 per 100,000 (95% CI: 0.5–0.8) to 1.6 per 100,000 (95% CI: 1.1–2.4), with an EAPC of 2.73 (95% CI: 2.57–2.90). Vietnam exhibited one of the most notable rises, with ASIR increasing from 1.3 per 100,000 (95% CI: 0.7–1.9) to 5.3 per 100,000 (95% CI: 2.9–7.9), and an EAPC of 5.20 (95% CI: 5.00–5.39). India also experienced a rise in ASIR, from 0.7 per 100,000 (95% CI: 0.5–0.9) to 1.5 per 100,000 (95% CI: 1.2–2.0), with an EAPC of 2.83 (95% CI: 2.73–2.93), while in Nepal, ASIR increased from 0.6 per 100,000 (95% CI: 0.3–1.0) to 1.3 per 100,000 (95% CI: 0.8–2.4), with an EAPC of 2.61 (95% CI: 2.51–2.71). These trends suggest a growing incidence of thyroid cancer across various socio-demographic contexts, posing challenges for early detection and risk factor mitigation, particularly in resource-limited settings.

### 3.3 Geographical distribution and trend analysis of the burden of endocrine-related cancers among reproductive-age women in the Asia-Pacific region

In 2021, the disease burden exhibited significant stratification driven by both cancer type and development level (**Table 1**).

**Table 1:** Regional disease burden of endocrine-related cancers in reproductive-age women in the Asia-Pacific region, 1999-2021.

Location	Cause	Measure	vNum_1990_CI	ASR_1990_CI	Num_2021_CI	ASR_2021_CI	EAPC_CI
Australia	Breast cancer	DALYs	21719.8 (20192.1–23395.7)	240.8 (223.9–259.4)	17763.3 (15523.6–20147.7)	145.9 (127.5–165.5)	-1.95 (-2.09 to -1.82)
Australia	Breast cancer	Deaths	424.3 (393.7–455.7)	4.7 (4.4–5.1)	327.9 (286.5–370.6)	2.7 (2.4–3)	-2.16 (-2.3 to -2.03)
Australia	Breast cancer	Incidence	2073.3 (1897.1–2249.9)	23 (21–24.9)	3083.7 (2623.2–3553.2)	25.3 (21.6–29.2)	0.16 (-0.13 to 0.46)
Bangladesh	Breast cancer	DALYs	43039.6 (30469.9–60742.6)	86.5 (61.3–122.1)	127603.5 (92793.4–172463)	137.3 (99.8–185.6)	1.47 (1.3 to 1.63)
Bangladesh	Breast cancer	Deaths	836.5 (593.9–1178.4)	1.7 (1.2–2.4)	2492.3 (1803.9–3399.5)	2.7 (1.9–3.7)	1.5 (1.34 to 1.67)
Bangladesh	Breast cancer	Incidence	1422 (1007.5–2010.9)	2.9 (2–4)	6946.8 (4986.5–9393.7)	7.5 (5.4–10.1)	3.18 (2.98 to 3.38)
China	Breast cancer	DALYs	699470.2 (554280.9–866932.8)	107.4 (85.1–133.2)	861866.6 (648610.2–1119103)	133.8 (100.7–173.8)	0.36 (0.23 to 0.5)
China	Breast cancer	Deaths	13566.9 (10747.4–16802.6)	2.1 (1.7–2.6)	16439.3 (12282.1–21419.6)	2.6 (1.9–3.3)	0.31 (0.17 to 0.46)
China	Breast cancer	Incidence	36688.9 (29142.1–45382.6)	5.6 (4.5–7)	110371.4 (82542.9–142344.8)	17.1 (12.8–22.1)	3.59 (3.5 to 3.67)
Fiji	Breast cancer	DALYs	1357.7 (1046.9–1746.4)	343.7 (265–442.1)	1982.8 (1411.2–2752.2)	429.7 (305.8–596.4)	0.95 (0.74 to 1.16)
Fiji	Breast cancer	Deaths	26.6 (20.6–34.4)	6.7 (5.2–8.7)	39.4 (27.9–54.2)	8.5 (6.1–11.8)	1.02 (0.81 to 1.23)
Fiji	Breast cancer	Incidence	61.2 (47.2–79.1)	15.5 (12–20)	86.7 (60.9–120.9)	18.8 (13.2–26.2)	0.59 (0.37 to 0.81)
India	Breast cancer	DALYs	425004.4 (368623.9–485953.8)	104.2 (90.4–119.1)	1161292.9 (999509.8–1360321.8)	151.9 (130.8–178)	1.18 (1.04 to 1.33)
India	Breast cancer	Deaths	8429.1 (7342.1–9642.6)	2.1 (1.8–2.4)	22806.4 (19651.4–26603.1)	3 (2.6–3.5)	1.15 (1 to 1.3)
India	Breast cancer	Incidence	15027.7 (13125.4–17173.9)	3.7 (3.2–4.2)	60765.1 (52296.5–71136)	7.9 (6.8–9.3)	2.5 (2.29 to 2.7)
Indonesia	Breast cancer	DALYs	142440.1 (102593.6–195070.8)	147.1 (105.9–201.4)	361952.4 (250192.2–513831.3)	237.9 (164.5–337.7)	1.57 (1.43 to 1.72)
Indonesia	Breast cancer	Deaths	2857 (2080.6–3896)	3 (2.1–4)	7330.1 (5048.3–10483.2)	4.8 (3.3–6.9)	1.63 (1.48 to 1.78)
Indonesia	Breast cancer	Incidence	5442.5 (3973.2–7426.8)	5.6 (4.1–7.7)	18805.7 (13093–26677.7)	12.4 (8.6–17.5)	2.55 (2.45 to 2.66)
Japan	Breast cancer	DALYs	93339.8 (89926.9–96902.8)	143.9 (138.6–149.4)	78163.5 (74117.1–82299.4)	155.6 (147.5–163.8)	0.14 (-0.02 to 0.29)
Japan	Breast cancer	Deaths	1792.1 (1749.4–1836)	2.8 (2.7–2.8)	1459 (1417.2–1494.1)	2.9 (2.8–3)	-0.01 (-0.17 to 0.15)
Japan	Breast cancer	Incidence	11233.5 (10285.6–12122.6)	17.3 (15.9–18.7)	14132.7 (12903.2–15316.9)	28.1 (25.7–30.5)	1.55 (1.3 to 1.81)
Malaysia	Breast cancer	DALYs	17644.6 (14730.4–20790.9)	194.9 (162.7–229.7)	48181.5 (38303.6–58958.4)	283 (225–346.3)	0.98 (0.82 to 1.15)
Malaysia	Breast cancer	Deaths	353.4 (295.1–418.1)	3.9 (3.3–4.6)	961 (764.8–1175.5)	5.6 (4.5–6.9)	0.97 (0.82 to 1.13)
Malaysia	Breast cancer	Incidence	808.4 (672.2–950.1)	8.9 (7.4–10.5)	3459.3 (2687.1–4233.4)	20.3 (15.8–24.9)	2.54 (2.41 to 2.66)
Mongolia	Breast cancer	DALYs	504.7 (397.4–625.2)	48.8 (38.4–60.5)	1352.5 (1050.4–1649.9)	79.3 (61.6–96.8)	1.46 (1.27 to 1.65)
Mongolia	Breast cancer	Deaths	10 (7.8–12.4)	1 (0.8–1.2)	27 (21.1–33)	1.6 (1.2–1.9)	1.55 (1.36 to 1.74)
Mongolia	Breast cancer	Incidence	18.9 (14.7–23.5)	1.8 (1.4–2.3)	77.9 (60.4–96.8)	4.6 (3.5–5.7)	3.16 (3 to 3.31)
Nepal	Breast cancer	DALYs	7187.9 (5013.8–9842.3)	77.9 (54.3–106.6)	17999.4 (12382.4–25182.1)	98.2 (67.6–137.4)	0.75 (0.51 to 0.99)
Nepal	Breast cancer	Deaths	143.7 (99.3–197)	1.6 (1.1–2.1)	354.1 (241.4–497.5)	1.9 (1.3–2.7)	0.7 (0.44 to 0.95)
Nepal	Breast cancer	Incidence	237.6 (164.5–327.6)	2.6 (1.8–3.5)	869.4 (594.5–1205.2)	4.7 (3.2–6.6)	2 (1.76 to 2.24)
New Zealand	Breast cancer	DALYs	5859.9 (5390.2–6305.6)	320.8 (295.1–345.2)	4800.3 (4415–5223.5)	198.8 (182.9–216.4)	-1.41 (-1.58 to -1.25)
New Zealand	Breast cancer	Deaths	114.7 (105.6–123.3)	6.3 (5.8–6.8)	91.2 (84.2–98.6)	3.8 (3.5–4.1)	-1.49 (-1.66 to -1.32)
New Zealand	Breast cancer	Incidence	532.2 (476.1–592.2)	29.1 (26.1–32.4)	683.2 (619–756.5)	28.3 (25.6–31.3)	0.15 (-0.08 to 0.37)
Republic of Korea	Breast cancer	DALYs	22101.8 (18988.2–26131.9)	86.6 (74.4–102.4)	33109.9 (27033.3–39822.3)	141.3 (115.4–170)	1.73 (1.5 to 1.95)
Republic of Korea	Breast cancer	Deaths	423.8 (363.2–503.1)	1.7 (1.4–2)	616.1 (505.6–734.3)	2.6 (2.2–3.1)	1.63 (1.4 to 1.86)
Republic of Korea	Breast cancer	Incidence	1373.5 (1155.5–1636.4)	5.4 (4.5–6.4)	5619.6 (4507.8–6854.1)	24 (19.2–29.3)	5.34 (4.8 to 5.87)
Singapore	Breast cancer	DALYs	3428.5 (3142–3769.6)	181.5 (166.4–199.6)	3706.1 (3329.8–4121.6)	126.1 (113.3–140.2)	-1.42 (-1.67 to -1.18)
Singapore	Breast cancer	Deaths	66.4 (60.9–72.7)	3.5 (3.2–3.9)	69.1 (62.6–76.6)	2.4 (2.1–2.6)	-1.6 (-1.85 to -1.34)
Singapore	Breast cancer	Incidence	269.6 (241.1–301)	14.3 (12.8–15.9)	609.6 (535.3–700.5)	20.7 (18.2–23.8)	1.11 (0.83 to 1.39)
Thailand	Breast cancer	DALYs	43657.8 (34853.6–53837.1)	135.1 (107.9–166.6)	91876.9 (69021.1–121406)	278.9 (209.5–368.6)	2.25 (1.85 to 2.66)
Thailand	Breast cancer	Deaths	867.5 (688.6–1055.1)	2.7 (2.1–3.3)	1810.3 (1354.8–2376.3)	5.5 (4.1–7.2)	2.26 (1.87 to 2.65)
Thailand	Breast cancer	Incidence	2227.1 (1746.1–2714.7)	6.9 (5.4–8.4)	8071.5 (5887.6–10700.6)	24.5 (17.9–32.5)	4.14 (3.72 to 4.57)
Viet Nam	Breast cancer	DALYs	28058.3 (20015–37819.5)	81.4 (58–109.7)	79158.8 (55710.8–110354.1)	153.4 (107.9–213.8)	1.94 (1.86 to 2.02)
Viet Nam	Breast cancer	Deaths	551.4 (394.5–740.5)	1.6 (1.1–2.1)	1566.9 (1098.5–2192.6)	3 (2.1–4.2)	1.99 (1.9 to 2.09)
Viet Nam	Breast cancer	Incidence	1232.4 (879.1–1679.5)	3.6 (2.5–4.9)	5978.7 (4230.5–8318.6)	11.6 (8.2–16.1)	3.77 (3.68 to 3.87)
Australia	Ovarian cancer	DALYs	4907.6 (4269.8–5382.9)	54.4 (47.3–59.7)	3150 (2796.4–3542.6)	25.9 (23–29.1)	-2.35 (-2.79 to -1.9)
Australia	Ovarian cancer	Deaths	96.6 (83.6–106.1)	1.1 (0.9–1.2)	62.3 (54.8–70.5)	0.5 (0.5–0.6)	-2.35 (-2.81 to -1.88)
Australia	Ovarian cancer	Incidence	304.6 (267.1–337.1)	3.4 (3–3.7)	225.5 (200.3–252.1)	1.9 (1.6–2.1)	-1.83 (-2.31 to -1.34)
Bangladesh	Ovarian cancer	DALYs	7303.7 (4778.6–11475.6)	14.7 (9.6–23.1)	24915.8 (12793.2–44061)	26.8 (13.8–47.4)	2.08 (1.98 to 2.17)
Bangladesh	Ovarian cancer	Deaths	137 (88.7–218)	0.3 (0.2–0.4)	472.2 (245.3–836.3)	0.5 (0.3–0.9)	2.13 (2.03 to 2.23)
Bangladesh	Ovarian cancer	Incidence	380 (245.5–598.2)	0.8 (0.5–1.2)	1528.2 (786–2715)	1.6 (0.8–2.9)	2.59 (2.51 to 2.68)
China	Ovarian cancer	DALYs	153874.3 (91308.5–209935.5)	23.6 (14–32.2)	176403.4 (126934.2–236354.7)	27.4 (19.7–36.7)	0.19 (0.06 to 0.32)
China	Ovarian cancer	Deaths	2913.1 (1760.6–3975.1)	0.4 (0.3–0.6)	3531.8 (2553.1–4734.9)	0.5 (0.4–0.7)	0.36 (0.23 to 0.5)
China	Ovarian cancer	Incidence	8587.9 (5020.1–11724.9)	1.3 (0.8–1.8)	11339.4 (8278.9–15199.5)	1.8 (1.3–2.4)	0.76 (0.65 to 0.87)
Fiji	Ovarian cancer	DALYs	59.9 (41.8–86.4)	15.2 (10.6–21.9)	88.8 (60.4–126.1)	19.2 (13.1–27.3)	0.76 (0.68 to 0.84)
Fiji	Ovarian cancer	Deaths	1.2 (0.8–1.7)	0.3 (0.2–0.4)	1.7 (1.2–2.5)	0.4 (0.3–0.5)	0.82 (0.72 to 0.92)
Fiji	Ovarian cancer	Incidence	4.6 (3.2–6.5)	1.2 (0.8–1.6)	7.3 (4.9–10.6)	1.6 (1.1–2.3)	0.92 (0.83 to 1.01)
India	Ovarian cancer	DALYs	73402.5 (52965.7–100866.4)	18 (13–24.7)	210107.1 (171622–247924.6)	27.5 (22.5–32.4)	1.28 (1.15 to 1.42)
India	Ovarian cancer	Deaths	1420.4 (1026.3–1958.7)	0.3 (0.3–0.5)	4077.1 (3327.4–4826.6)	0.5 (0.4–0.6)	1.29 (1.14 to 1.43)
India	Ovarian cancer	Incidence	3769.1 (2714.9–5253)	0.9 (0.7–1.3)	12322.2 (10099.5–14760.5)	1.6 (1.3–1.9)	1.72 (1.59 to 1.84)
Indonesia	Ovarian cancer	DALYs	27920.9 (18190.9–54492)	28.8 (18.8–56.3)	76261.1 (47714.5–121214.4)	50.1 (31.4–79.7)	1.76 (1.62 to 1.91)
Indonesia	Ovarian cancer	Deaths	521.4 (343.5–1009.5)	0.5 (0.4–1)	1461.4 (914.4–2325.7)	1 (0.6–1.5)	1.88 (1.74 to 2.02)
Indonesia	Ovarian cancer	Incidence	2162.3 (1365.6–4370.4)	2.2 (1.4–4.5)	6612.3 (4107.3–10276.7)	4.3 (2.7–6.8)	2.04 (1.85 to 2.22)
Japan	Ovarian cancer	DALYs	35607.4 (34719.6–36461.7)	54.9 (53.5–56.2)	22989.7 (22318.7–23655.9)	45.8 (44.4–47.1)	-0.49 (-0.66 to -0.32)
Japan	Ovarian cancer	Deaths	719.7 (702.7–736.6)	1.1 (1.1–1.1)	467.7 (454.8–481.7)	0.9 (0.9–1)	-0.49 (-0.68 to -0.3)
Japan	Ovarian cancer	Incidence	1892 (1803.9–1983.4)	2.9 (2.8–3.1)	1551.1 (1464.5–1657.6)	3.1 (2.9–3.3)	0.27 (0.08 to 0.46)
Malaysia	Ovarian cancer	DALYs	1648.4 (1168.2–2990.1)	18.2 (12.9–33)	4848 (3544.6–7803.2)	28.5 (20.8–45.8)	1.58 (1.13 to 2.03)
Malaysia	Ovarian cancer	Deaths	31.7 (22.5–55.7)	0.4 (0.2–0.6)	94.1 (69.3–147.8)	0.6 (0.4–0.9)	1.61 (1.15 to 2.07)
Malaysia	Ovarian cancer	Incidence	129.4 (91–250.7)	1.4 (1–2.8)	446.3 (324.9–742.3)	2.6 (1.9–4.4)	2.11 (1.61 to 2.61)
Mongolia	Ovarian cancer	DALYs	320.3 (211.5–469.2)	31 (20.5–45.4)	795 (553.2–1069.9)	46.6 (32.4–62.7)	1.33 (1.21 to 1.45)
Mongolia	Ovarian cancer	Deaths	6.1 (4.1–8.8)	0.6 (0.4–0.9)	15.8 (11–21.1)	0.9 (0.6–1.2)	1.55 (1.44 to 1.66)
Mongolia	Ovarian cancer	Incidence	15.5 (10.1–22.7)	1.5 (1–2.2)	41 (28.4–56)	2.4 (1.7–3.3)	1.56 (1.44 to 1.68)
Nepal	Ovarian cancer	DALYs	1370.8 (767.2–2352.3)	14.9 (8.3–25.5)	4720.8 (3010.6–8593.1)	25.8 (16.4–46.9)	1.79 (1.74 to 1.85)
Nepal	Ovarian cancer	Deaths	26.4 (14.8–45.2)	0.3 (0.2–0.5)	90.1 (56.9–163)	0.5 (0.3–0.9)	1.75 (1.69 to 1.82)
Nepal	Ovarian cancer	Incidence	69.1 (39.5–119.6)	0.7 (0.4–1.3)	277.1 (174.1–503.2)	1.5 (1–2.7)	2.29 (2.26 to 2.31)
New Zealand	Ovarian cancer	DALYs	884 (792.6–999.5)	48.4 (43.4–54.7)	665.4 (574.1–768.6)	27.6 (23.8–31.8)	-1.94 (-2.16 to -1.71)
New Zealand	Ovarian cancer	Deaths	17.3 (15.5–19.7)	0.9 (0.8–1.1)	13.2 (11.3–15.4)	0.5 (0.5–0.6)	-1.89 (-2.12 to -1.66)
New Zealand	Ovarian cancer	Incidence	61.7 (54.6–70.5)	3.4 (3–3.9)	49.3 (42.3–57.2)	2 (1.8–2.4)	-1.77 (-2 to -1.54)
Republic of Korea	Ovarian cancer	DALYs	6973.2 (4616.4–8005.2)	27.3 (18.1–31.4)	8475.9 (6232.5–10252.8)	36.2 (26.6–43.8)	0.95 (0.84 to 1.07)
Republic of Korea	Ovarian cancer	Deaths	130.7 (90–150)	0.5 (0.4–0.6)	170.9 (125.4–208.5)	0.7 (0.5–0.9)	1.22 (1.1 to 1.34)
Republic of Korea	Ovarian cancer	Incidence	367.4 (234.8–427)	1.4 (0.9–1.7)	573.5 (416.2–703)	2.4 (1.8–3)	1.78 (1.65 to 1.91)
Singapore	Ovarian cancer	DALYs	845.7 (761.4–941.1)	44.8 (40.3–49.8)	958 (845.9–1079.9)	32.6 (28.8–36.7)	-1.33 (-1.61 to -1.04)
Singapore	Ovarian cancer	Deaths	16.5 (14.8–18.5)	0.9 (0.8–1)	19.3 (16.9–22)	0.7 (0.6–0.7)	-1.26 (-1.55 to -0.97)
Singapore	Ovarian cancer	Incidence	44.4 (39.5–49.3)	2.4 (2.1–2.6)	64.7 (56.6–74)	2.2 (1.9–2.5)	-0.44 (-0.75 to -0.14)
Thailand	Ovarian cancer	DALYs	9089.3 (6505.6–11784.5)	28.1 (20.1–36.5)	20061.9 (10586.2–28070.8)	60.9 (32.1–85.2)	2.54 (2.29 to 2.79)
Thailand	Ovarian cancer	Deaths	172.2 (123.3–223.6)	0.5 (0.4–0.7)	388.7 (204.9–550.2)	1.2 (0.6–1.7)	2.66 (2.42 to 2.9)
Thailand	Ovarian cancer	Incidence	758.8 (530.9–990.2)	2.3 (1.6–3.1)	1952 (1016.4–2734.2)	5.9 (3.1–8.3)	2.96 (2.65 to 3.27)
Viet Nam	Ovarian cancer	DALYs	3233.7 (2213.8–4449.3)	9.4 (6.4–12.9)	10112.6 (6343.8–14743.8)	19.6 (12.3–28.6)	2.26 (2.1 to 2.42)
Viet Nam	Ovarian cancer	Deaths	60.7 (41.6–84.3)	0.2 (0.1–0.2)	199.2 (125.1–298.5)	0.4 (0.2–0.6)	2.46 (2.28 to 2.64)
Viet Nam	Ovarian cancer	Incidence	261.4 (178.1–367.6)	0.8 (0.5–1.1)	911.8 (584.5–1328.4)	1.8 (1.1–2.6)	2.57 (2.39 to 2.75)
Australia	Thyroid cancer	DALYs	237.4 (189.2–297)	2.6 (2.1–3.3)	307.6 (211.2–438.1)	2.5 (1.7–3.6)	0.46 (-0.37 to 1.29)
Australia	Thyroid cancer	Deaths	3.3 (2.7–3.9)	0 (0–0)	3.3 (2.4–4.3)	0 (0–0)	-0.48 (-1.22 to 0.27)
Australia	Thyroid cancer	Incidence	135.2 (107.3–165.6)	1.5 (1.2–1.8)	298.7 (211.3–407.3)	2.5 (1.7–3.3)	2.17 (1.15 to 3.2)
Bangladesh	Thyroid cancer	DALYs	3547.3 (2236.8–5412.7)	7.1 (4.5–10.9)	6010.1 (3265.8–13448.2)	6.5 (3.5–14.5)	0.01 (-0.21 to 0.22)
Bangladesh	Thyroid cancer	Deaths	58.9 (37.2–90)	0.1 (0.1–0.2)	95.7 (52.8–211)	0.1 (0.1–0.2)	-0.12 (-0.33 to 0.08)
Bangladesh	Thyroid cancer	Incidence	328.6 (196.3–509.3)	0.7 (0.4–1)	1337.9 (720.5–2941.8)	1.4 (0.8–3.2)	2.98 (2.73 to 3.23)
China	Thyroid cancer	DALYs	23020.2 (16641–29757.9)	3.5 (2.6–4.6)	17517.1 (12021.4–27142.2)	2.7 (1.9–4.2)	-1.24 (-1.4 to -1.08)
China	Thyroid cancer	Deaths	397.4 (284.2–507.5)	0.1 (0–0.1)	250.9 (179–388.4)	0 (0–0.1)	-1.88 (-2.06 to -1.71)
China	Thyroid cancer	Incidence	4323.8 (3026–5485.8)	0.7 (0.5–0.8)	10016.6 (7206.9–15596.1)	1.6 (1.1–2.4)	2.73 (2.57 to 2.9)
Fiji	Thyroid cancer	DALYs	25 (15.7–36.5)	6.3 (4–9.2)	30.4 (16.5–47.8)	6.6 (3.6–10.4)	0.2 (0.1 to 0.3)
Fiji	Thyroid cancer	Deaths	0.4 (0.3–0.6)	0.1 (0.1–0.2)	0.5 (0.3–0.8)	0.1 (0.1–0.2)	0.12 (0 to 0.23)
Fiji	Thyroid cancer	Incidence	4.6 (2.8–6.8)	1.2 (0.7–1.7)	8.2 (4.2–13.2)	1.8 (0.9–2.9)	1.26 (1.16 to 1.35)
India	Thyroid cancer	DALYs	26498.8 (19839.6–38274.6)	6.5 (4.9–9.4)	56439.8 (42900–75529.1)	7.4 (5.6–9.9)	0.43 (0.31 to 0.55)
India	Thyroid cancer	Deaths	446.6 (337.6–642.2)	0.1 (0.1–0.2)	928.2 (714.1–1218)	0.1 (0.1–0.2)	0.37 (0.26 to 0.48)
India	Thyroid cancer	Incidence	2725 (2065.6–3872.3)	0.7 (0.5–0.9)	11727.2 (9021.8–15387.6)	1.5 (1.2–2)	2.83 (2.73 to 2.93)
Indonesia	Thyroid cancer	DALYs	5224.8 (3183.2–6624.3)	5.4 (3.3–6.8)	8499 (5213.3–12271.1)	5.6 (3.4–8.1)	0.11 (-0.04 to 0.25)
Indonesia	Thyroid cancer	Deaths	93.8 (56.8–118.4)	0.1 (0.1–0.1)	150.3 (92.5–215.6)	0.1 (0.1–0.1)	0.11 (-0.04 to 0.26)
Indonesia	Thyroid cancer	Incidence	770.5 (467.4–975.7)	0.8 (0.5–1)	2332.5 (1470.8–3438.5)	1.5 (1–2.3)	1.99 (1.83 to 2.15)
Japan	Thyroid cancer	DALYs	1939.5 (1679.1–2249.3)	3 (2.6–3.5)	1600.7 (1306.2–1982.9)	3.2 (2.6–3.9)	0.1 (-0.02 to 0.23)
Japan	Thyroid cancer	Deaths	25.4 (24.5–26.3)	0 (0–0)	16.4 (15.7–17)	0 (0–0)	-0.79 (-0.98 to -0.6)
Japan	Thyroid cancer	Incidence	1418.2 (1250.6–1602.4)	2.2 (1.9–2.5)	1628.2 (1397.6–1846.1)	3.2 (2.8–3.7)	1.2 (1.02 to 1.38)
Malaysia	Thyroid cancer	DALYs	551.2 (347.2–744.2)	6.1 (3.8–8.2)	1041.3 (716.6–1404)	6.1 (4.2–8.2)	-0.1 (-0.39 to 0.18)
Malaysia	Thyroid cancer	Deaths	9.2 (6.1–12.4)	0.1 (0.1–0.1)	15.9 (11.2–20.9)	0.1 (0.1–0.1)	-0.42 (-0.68 to -0.16)
Malaysia	Thyroid cancer	Incidence	117.4 (72.6–160.6)	1.3 (0.8–1.8)	462.7 (307.8–649.1)	2.7 (1.8–3.8)	2.31 (2.13 to 2.49)
Mongolia	Thyroid cancer	DALYs	47.7 (27–80.1)	4.6 (2.6–7.7)	84.7 (51.6–130.4)	5 (3–7.6)	-0.2 (-0.74 to 0.34)
Mongolia	Thyroid cancer	Deaths	0.9 (0.5–1.4)	0.1 (0–0.1)	1.5 (0.9–2.4)	0.1 (0.1–0.1)	-0.24 (-0.75 to 0.28)
Mongolia	Thyroid cancer	Incidence	5.4 (3.1–8.8)	0.5 (0.3–0.9)	19.3 (11.9–30.4)	1.1 (0.7–1.8)	2.5 (1.92 to 3.09)
Nepal	Thyroid cancer	DALYs	635.5 (348.8–1038)	6.9 (3.8–11.2)	1311.1 (761.6–2400.7)	7.2 (4.2–13.1)	0.18 (0.04 to 0.33)
Nepal	Thyroid cancer	Deaths	10.7 (6–17.6)	0.1 (0.1–0.2)	21.6 (12.8–39.2)	0.1 (0.1–0.2)	0.11 (-0.06 to 0.27)
Nepal	Thyroid cancer	Incidence	57.2 (31.1–94.8)	0.6 (0.3–1)	247.3 (143.4–444.4)	1.3 (0.8–2.4)	2.61 (2.51 to 2.71)
New Zealand	Thyroid cancer	DALYs	36.1 (27.6–47.3)	2 (1.5–2.6)	29.6 (21–40.5)	1.2 (0.9–1.7)	-1.16 (-2.47 to 0.17)
New Zealand	Thyroid cancer	Deaths	0.6 (0.5–0.8)	0 (0–0)	0.4 (0.3–0.5)	0 (0–0)	-1.65 (-2.94 to -0.34)
New Zealand	Thyroid cancer	Incidence	14.3 (10.6–19.3)	0.8 (0.6–1.1)	18.6 (13.3–25.1)	0.8 (0.6–1)	0.57 (-0.76 to 1.92)
Republic of Korea	Thyroid cancer	DALYs	899.5 (553.5–1649)	3.5 (2.2–6.5)	1033.2 (613.1–1713.1)	4.4 (2.6–7.3)	1.82 (0.8 to 2.85)
Republic of Korea	Thyroid cancer	Deaths	14.1 (9–25.3)	0.1 (0–0.1)	11 (6.9–17.6)	0 (0–0.1)	0.41 (-0.52 to 1.34)
Republic of Korea	Thyroid cancer	Incidence	345.1 (220.1–607.8)	1.4 (0.9–2.4)	1003.5 (625.4–1558.7)	4.3 (2.7–6.7)	4.93 (3.58 to 6.3)
Singapore	Thyroid cancer	DALYs	62.1 (50.5–77.4)	3.3 (2.7–4.1)	55 (40.6–74.2)	1.9 (1.4–2.5)	-1.66 (-2.28 to -1.03)
Singapore	Thyroid cancer	Deaths	0.9 (0.7–1.1)	0 (0–0.1)	0.6 (0.5–0.8)	0 (0–0)	-2.65 (-3.22 to -2.08)
Singapore	Thyroid cancer	Incidence	31.9 (25.5–38.9)	1.7 (1.4–2.1)	51.2 (39.1–66.9)	1.7 (1.3–2.3)	0.34 (-0.42 to 1.12)
Thailand	Thyroid cancer	DALYs	1572.5 (1109.1–2287.9)	4.9 (3.4–7.1)	1884.7 (1201.3–3181.3)	5.7 (3.6–9.7)	-0.23 (-0.83 to 0.37)
Thailand	Thyroid cancer	Deaths	26.8 (18.8–39)	0.1 (0.1–0.1)	28.2 (17.9–46.1)	0.1 (0.1–0.1)	-0.6 (-1.15 to -0.04)
Thailand	Thyroid cancer	Incidence	388.6 (273.6–563.2)	1.2 (0.8–1.7)	1025.1 (647.4–1721.2)	3.1 (2–5.2)	2.3 (1.57 to 3.04)
Viet Nam	Thyroid cancer	DALYs	2163.7 (1236.9–3192.9)	6.3 (3.6–9.3)	5817.9 (3198.8–8832.9)	11.3 (6.2–17.1)	2.43 (2.16 to 2.69)
Viet Nam	Thyroid cancer	Deaths	35.5 (21.5–52.2)	0.1 (0.1–0.2)	87.8 (50.5–134.7)	0.2 (0.1–0.3)	2.16 (1.92 to 2.41)
Viet Nam	Thyroid cancer	Incidence	434.1 (248.1–664.3)	1.3 (0.7–1.9)	2734 (1485.6–4051.9)	5.3 (2.9–7.9)	5.2 (5 to 5.39)

Breast Cancer: Significant disparities were observed. China reported the highest absolute incidence (110,371.4 new cases), whereas Mongolia had the lowest (77.9 cases). Mortality followed a similar pattern, with India recording the highest number of deaths (22,806.4). Trend analysis revealed a distinct SDI gradient: High-SDI countries like New Zealand showed high incidence but declining mortality (negative EAPCs). Conversely, Low-SDI countries demonstrated upward trends in both metrics; notably, Vietnam recorded a high EAPC in ASIR of 3.77, and Thailand showed an increasing mortality trend (EAPC: 2.26).

Ovarian Cancer: India bore the heaviest burden, reporting 12,322.2 new cases and 4,077.1 deaths. Trend analysis showed a divergence: High-SDI countries (e.g., Australia, EAPC: –2.35) continued to exhibit declining mortality, whereas Medium-to-High SDI countries like Thailand faced increasing DALY rates (EAPC: 2.66), indicating a rising disease burden (**Supplementary Figure S2**).

**Figure 4:**
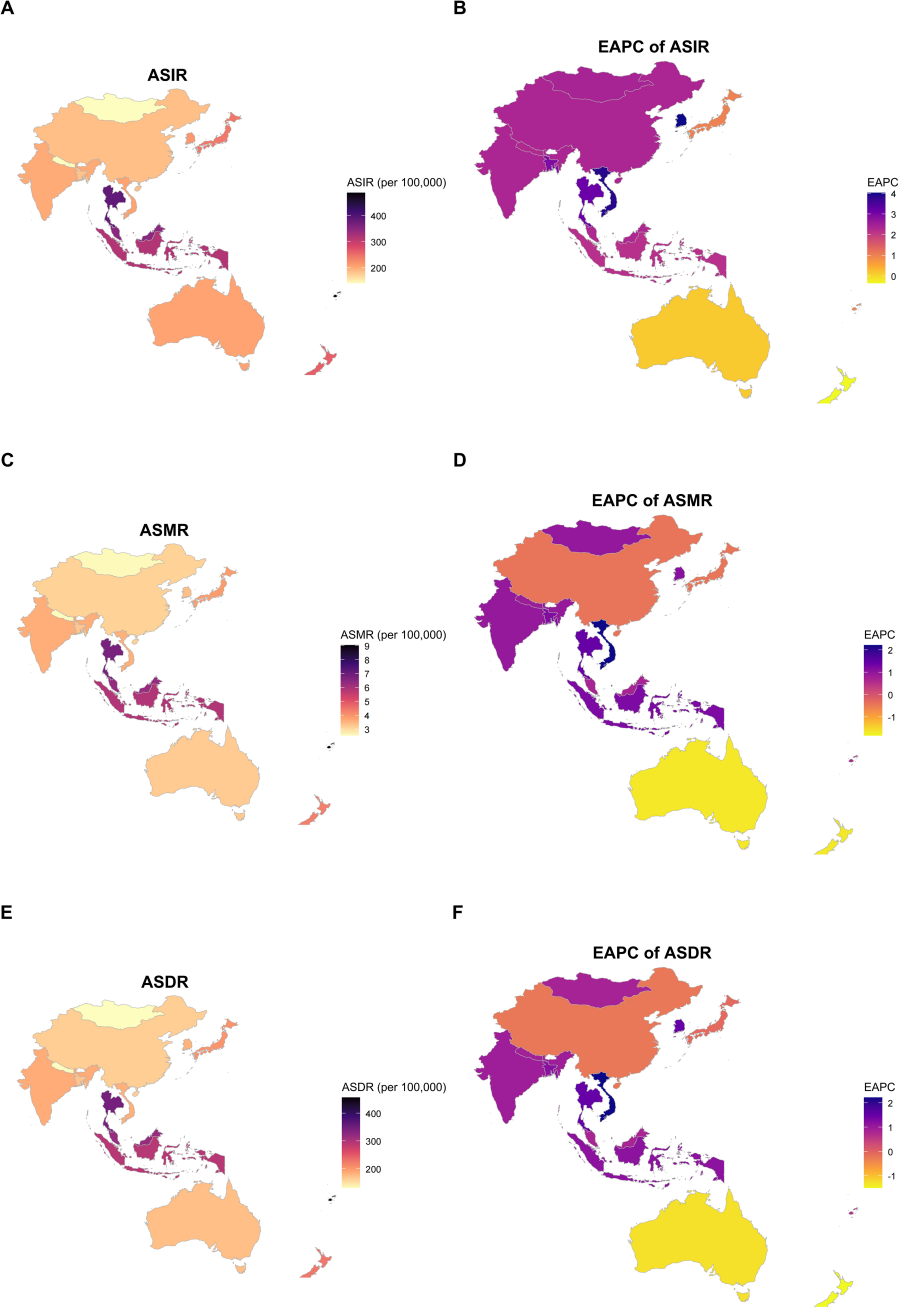
Geographical distribution of ASR and EAPC of incidence, mortality, and DALY for endocrine-related cancers in 2021. Note: Visualization showing the levels of current ASIR, ASMR, and ASDR (left) and EAPC (right) for 15 countries. Geographical distribution of ASR and EAPC for endocrine-related cancers in reproductive-age women. The color scale represents the absolute ASR value (magma color scheme) or the direction of the EAPC trend (plasma color scheme). Positive EAPC values indicate an increase in disease burden over time, while negative values ​​indicate a decreasing trend in disease burden. **(A)** ASIR of endocrine-related cancers in reproductive-age women in 15 Asia-Pacific countries in 2021. **(B)** EAPC of ASIR for endocrine-related cancers in reproductive-age women in 15 Asia-Pacific countries from 1990 to 2021. **(C)** ASMR of endocrine-related cancers in reproductive-age women in 15 Asia-Pacific countries in 2021. **(D)** EAPC of ASMR for endocrine-related cancers in reproductive-age women in 15 Asia-Pacific countries from 1990 to 2021. **(E)** ASDR of endocrine-related cancers in reproductive-age women in 15 Asia-Pacific countries in 2021. **(F)** EAPC of ASDR for endocrine-related cancers in reproductive-age women in 15 Asia-Pacific countries from 1990 to 2021.

Thyroid Cancer: India again reported the highest absolute burden (11,727.2 new cases). The most pronounced trends were observed in the disparity between SDI levels. High-SDI nations like Australia and Japan have reduced ASMR to near-zero levels, reflecting the effectiveness of early detection and clinical management strategies. In contrast, Low-SDI countries like Vietnam experienced marked increases in both incidence (EAPC: 2.83) and mortality (EAPC: 2.16), highlighting the urgent need for targeted interventions in these regions (**Figure 4**).

**Figure 5:**
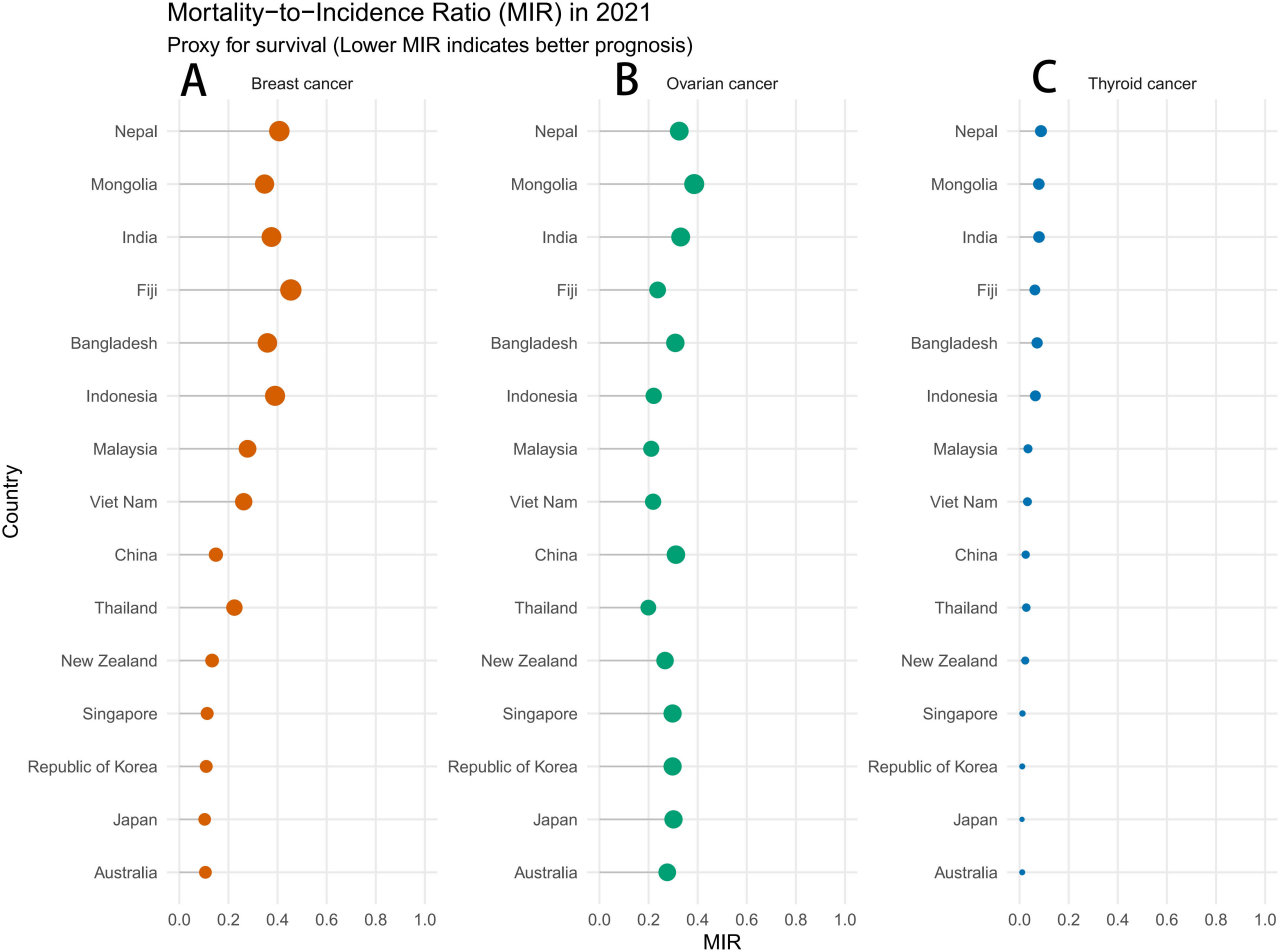
MIR of endocrine-related cancers in reproductive-age women in the Asia-Pacific region. **(A)** Breast cancer; **(B)** Ovarian cancer; **(C)** Thyroid cancer. Note: The MIR serves as a proxy for 5-year survival and quality of care. Values range from 0 to 1. A lower MIR (closer to 0) indicates favorable prognosis and effective management, while a higher MIR (closer to 1) suggests poor survival outcomes.

Survival and Healthcare Quality Assessment: To systematically evaluate the disparity in cancer prognosis across these regions, we calculated the Mortality-to-Incidence Ratio (MIR) for 2021 (**Figure 5**). The lollipop chart exposes a striking gradient in survival outcomes. High-SDI nations such as Australia and South Korea maintained MIRs below 0.2 for thyroid and breast cancers, reflecting superior early detection and treatment efficacy. In stark contrast, Low-to-Middle SDI countries like Fiji and Thailand recorded significantly higher MIRs (approaching 0.4–0.5 for breast cancer). This disparity highlights critical gaps in clinical management and underscores the urgent need to improve survival rates in these resource-limited settings.

### 3.4 Comparative analysis of risk factor patterns for endocrine-related cancers in 1990 and 2021

**Figure 6:**
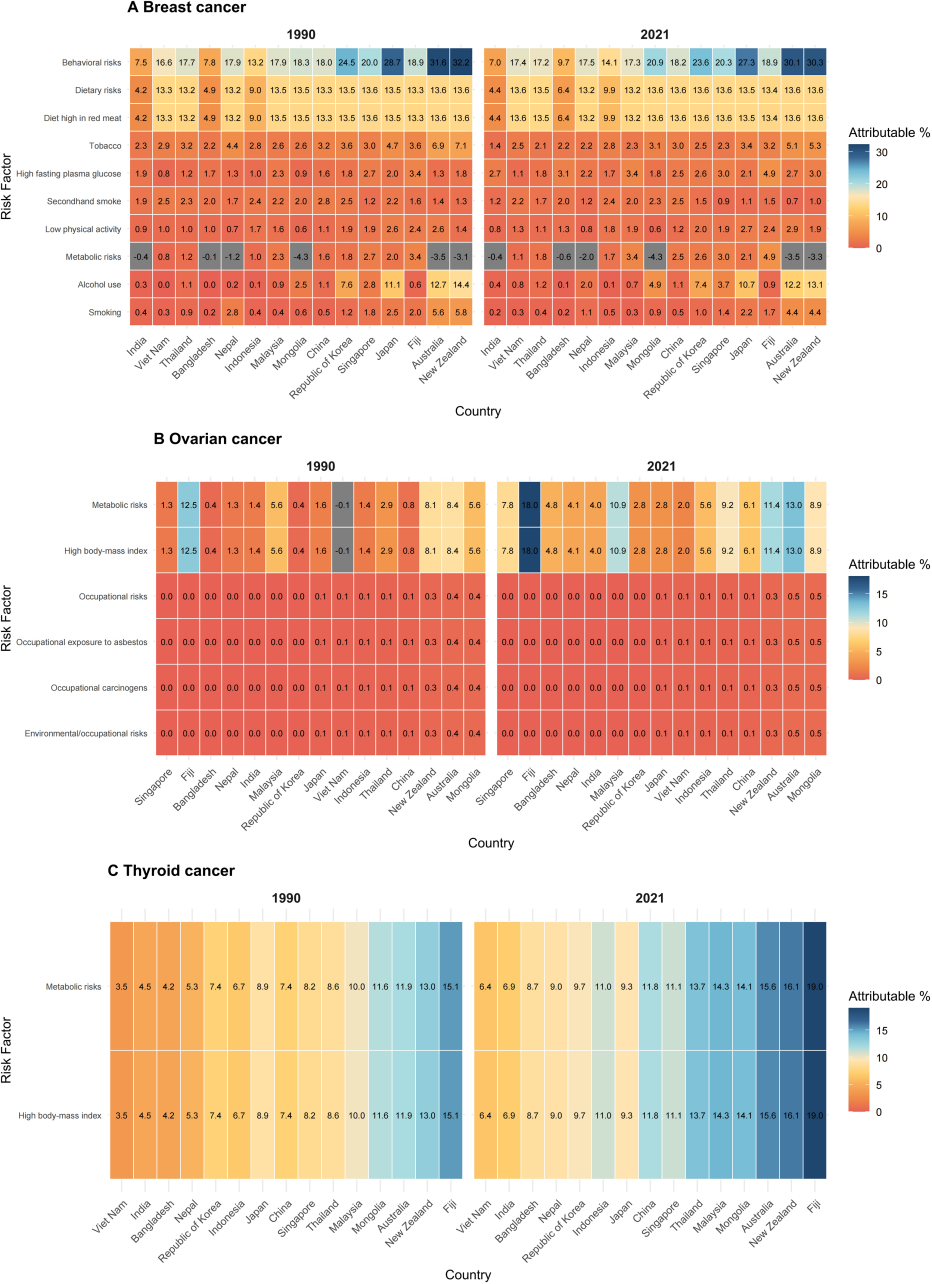
Major attributable risk factors and their percentage contributions to endocrine-related cancer deaths among reproductive-age women in the Asia-Pacific region.**(A)** breast cancer, **(B)** ovarian cancer, and **(C)** thyroid cancer. The heatmaps display the PAFs, representing the proportion of deaths that would be eliminated if the risk factor were removed. The color scale represents the magnitude of the attributable burden; darker colors indicate a higher percentage contribution. “Metabolic risks” is a composite cluster primarily including high body-mass index (BMI) and high fasting plasma glucose.

Using GBD 2021 data, this study systematically assessed the leading attributable risk factors and their proportional contributions to endocrine-related cancer mortality among reproductive-age women in the Asia-Pacific region. Behavioral risk factors—including smoking, alcohol consumption, and high sugar intake—are key drivers of breast cancer mortality, with their distribution showing a marked gradient across SDI levels. High-SDI countries have significantly reduced the proportional contributions of traditional behavioral risks. However, with the increasing prevalence of the Western lifestyle and the increased availability of processed foods, high sugar intake has emerged as a significant risk factor. For instance, the proportional contribution of high sugar intake to breast cancer mortality in South Korea rose from 8.2% in 1990 to 12.7% in 2021, highlighting the emerging contradiction between economic development and evolving health risks. In contrast, behavioral risk factors in low-SDI countries have shown limited improvement, thereby continuing to exacerbate the cancer burden (**Figure 6**).

Metabolic risk factors—including high body mass index (BMI) and diabetes—represent shared contributors to cancer burden across all SDI strata (18), with prevalence trends characterized by stable growth in high-SDI countries and accelerated increases in low-SDI countries. These factors are particularly significant in contributing to ovarian cancer mortality (19). In high-SDI countries, despite rising BMI levels—for instance, obesity rates among reproductive-age women in Australia have steadily increased (20)—diabetes screening programs, policies promoting physical activity, and comprehensive chronic disease management systems have partially offset the adverse effects of metabolic risk on mortality. In contrast, low-SDI countries such as India have experienced rapid nutritional transitions and urbanization, which have contributed to a more pronounced rise in obesity prevalence. Combined with limited health resources and inadequate metabolic risk management, these factors have intensified mortality associated with endocrine-related cancers.

In addition, occupational exposures—such as contact with chemicals and agricultural hazards—and environmental pollutants—including air pollution and endocrine-disrupting chemicals—contribute substantially to ovarian cancer mortality in low-SDI countries. Although high-SDI countries have significantly reduced occupational exposure risks through policies such as the EU REACH regulation (21), agricultural and industrial workers in low-SDI countries remain highly exposed. Moreover, the cancer burden attributable to environmental pollution is particularly severe in developing countries such as India and Bangladesh. Although the overall attributable risk of thyroid cancer mortality is relatively low, metabolic risk factors—including abnormal iodine intake, autoimmune thyroid disorders, and high BMI—remain significant contributors. In high-SDI countries such as Australia, metabolic risks contribute 15.6% to thyroid cancer mortality, which may be associated with improved diagnostic practices and more comprehensive management of advanced-stage disease. Conversely, in low-SDI countries such as Fiji, the contribution is higher (19%), reflecting persistent gaps in the prevention and control of underlying metabolic disorders.

### 3.5 Diverging future trends of endocrine-related cancers and implications for regional cancer control

**Figure 7:**
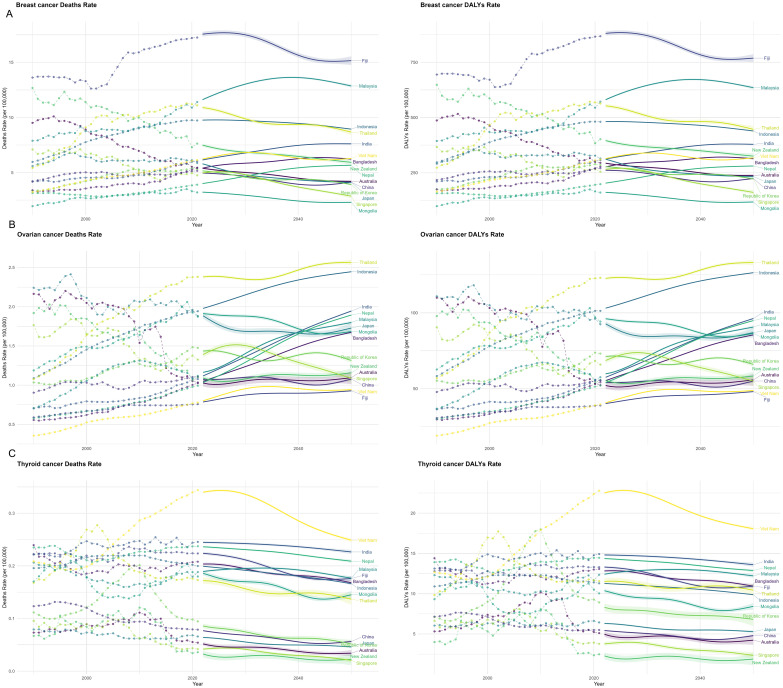
Predicting the burden of endocrine-related cancers in reproductive-age women in the Asia-Pacific region (1990-2050). **(A)** Breast cancer; **(B)** Ovarian cancer; **(C)** Thyroid cancer. Note: Dashed lines and dots represent observed mortality and DALY rates from the GBD study (1990-2021). Solid lines represent predicted results based on the GAM model, and the shaded areas surrounding the solid lines represent the 95% CIs of the projection. Labels indicate the predicted mortality and DALY rates for each country in 2050.

From 1990 to 2021, mortality rates and DALYs associated with endocrine-related cancers among reproductive-age women in the Asia-Pacific region exhibited substantial variation across countries, largely reflecting differences in socioeconomic development levels; future trajectories are projected to diverge even further (**Figure 7**).

Breast cancer has consistently remained the leading cause of cancer-related mortality among reproductive-age women (22). In Fiji, breast cancer mortality and DALY rates have consistently ranked among the highest in the Asia-Pacific region, with a marked increase observed after 2005. Although a slight decline is projected after 2050, the overall improvement is expected to remain limited. In high-SDI countries—including Japan, South Korea, Singapore, and China—breast cancer mortality and DALY rates have shown a steady downward trend. In medium-SDI countries—such as Indonesia, Thailand, and Malaysia—both indicators are projected to decline slightly by 2050, although the decrease is expected to be less pronounced than that observed in high-SDI countries. In low-SDI countries, although some improvements are anticipated, the magnitude of change is expected to be minimal, and the burden of breast cancer mortality and DALYs will remain substantial. .

Ovarian cancer, the most lethal form of gynecologic malignancy (7), exhibits a clear upward trend in mortality and DALY rates in countries and regions with medium to low SDI. By 2050, Thailand and Indonesia are projected to bear the highest ovarian cancer mortality and DALY burdens in the Asia-Pacific region. The burden in Malaysia, Mongolia, and Bangladesh is expected to remain relatively stable over the coming decades. In high-SDI countries—such as South Korea, New Zealand, and Australia—ovarian cancer mortality and DALY rates have exhibited a gradual downward trend.

Owing to its inherent biological characteristics, thyroid cancer has consistently been associated with low mortality and DALY rates among reproductive-age women in the Asia-Pacific region (23). However, in low-SDI countries and regions—such as Vietnam, Thailand, and Bangladesh—thyroid cancer mortality and DALY rates are projected to rise, with Vietnam expected to experience particularly marked increases over the coming decades. In contrast, high-SDI countries—such as China, Singapore, Australia, and New Zealand—are projected to experience a gradual decline in thyroid cancer mortality and DALY burdens.

reproductive-age women.

## 4 Discussion

Between 1990 and 2021, breast cancer, ovarian cancer, and thyroid cancer have become the three most prevalent endocrine-related tumors among reproductive-age women in the Asia-Pacific region (24). Their epidemiological patterns exhibit tumor-specific characteristics, country-specific heterogeneity, and inequalities derived from the SDI.

### 4.1 Tumor-specific and country-level disparities in breast cancer burden

Breast cancer has long constituted the predominant cause of mortality among reproductive-age women (22). In high-SDI countries, such as Japan, Singapore, and South Korea, where lifestyles have become increasingly Westernized—characterized by high-fat diets, reduced physical activity, delayed childbearing, and rising use of hormone replacement therapy and oral contraceptives—ASIR have consistently risen, showing a gradual upward trend (25, 26). Meanwhile, countries such as Australia have enhanced access to early detection and standardized treatment through screening systems that combine regular mammography with breast ultrasound (27, 28), whereas Japan has integrated HER2-targeted drugs into its healthcare system and established standardized treatment pathways, significantly improving the availability of early detection and treatment (29). As a result, ASMR in high-SDI countries has steadily declined, with five-year survival rates generally exceeding 80%, establishing a “high burden-low mortality” ‘controllable’ breast cancer management model. However, middle-to-high-SDI countries, such as China and Malaysia, face a paradoxical situation of “rapidly rising incidence rates and lagging declines in mortality rates.” Urbanization has led to an annual increase of 3–5% in the ASIR, but due to insufficient early screening coverage and delayed reimbursement for targeted drugs, over 60% of patients are diagnosed at more advanced stages, leading to a significantly lower decline in ASMR compared to high-SDI countries. The five-year survival rate gap between urban and rural areas exceeds 20%. In resource-scarce regions of low-SDI countries, patients often discontinue standardized treatment due to economic burdens, resulting in over 70% of cases being diagnosed at advanced stages, which creates a vicious cycle of “diagnosis at end-stage” and highlights structural imbalances in resource allocation (24, 30, 31). Therefore, for these resource-constrained settings, replicating the mammography-based screening models of high-income nations is impractical. The priority must shift towards a “resource-stratified adaptation” strategy: promoting Clinical Breast Examination (CBE) as a cost-effective primary screening tool and ensuring the availability of basic pathology services and essential medicines (e.g., tamoxifen). Prioritizing these fundamental interventions over expensive targeted therapies offers the most realistic pathway to reducing mortality under limited budgets.

### 4.2 The “low incidence–high mortality” paradox of ovarian cancer across SDI strata

Ovarian cancer is the deadliest type of gynecological malignancy (19). The global incidence pattern exhibits a striking “low incidence–high mortality” paradox, with regional disparities far surpassing those of breast cancer. Addressing this requires distinct strategies tailored to socioeconomic capacity.

In High-SDI nations, the focus has successfully shifted toward a “Precision-Prevention-Treatment” ecosystem. Primary prevention leverages genetic screening (covering ~30% of high-risk individuals) and prophylactic salpingo-oophorectomy, reducing risk by up to 70% (32–35). Secondary detection combines transvaginal ultrasound with the HE4-ROMA algorithm(Human Epididymis Protein 4-Risk of Ovarian Malignancy Algorithm), raising stage I detection rates to 45% (36, 37). Tertiary treatment now includes standardized cytoreductive surgery (R0 resection rates >80%) and PARP inhibitors, extending progression-free survival significantly (36–38). Notably, South Korea’s 2022 discontinuation of CA125(Cancer Antigen 125) screening for asymptomatic women marks a strategic pivot from “blind expansion” to “quality control,” prioritizing the reduction of false positives and overdiagnosis.

In Middle-SDI nations, the primary challenge is the “Capacity-Demand Mismatch.” As incidence rises with urbanization, infrastructure lags. For instance, in China, while urban incidence mirrors High-SDI nations, only 7% of primary care facilities possess transvaginal ultrasound capabilities. This structural deficit leads to an average diagnosis delay of 8.2 months, with neoadjuvant chemotherapy utilization below 30% (38, 39). The resulting urban-rural survival gap (18 percentage points) highlights that simply importing advanced treatments without bolstering primary diagnostic capacity is ineffective (40).

For Low-to-Middle SDI nations, facing the “triple burden” of rising incidence, mortality, and system fragility, replicating the high-cost Western model is unsustainable. Instead, we propose a “Resource-Adapted Sequential Strategy” focusing on two high-value, low-cost interventions: firstly, Opportunistic Primary Prevention: Instead of dedicated genetic screening programs, health systems should prioritize opportunistic salpingo-oophorectomy during other obstetric/gynecological procedures (e.g., cesarean sections or tubal sterilizations) (35, 41). This intervention adds negligible cost but provides permanent risk reduction, leveraging the high volume of C-sections in many developing regions. Secondly, Symptom-Triggered Triage (The “Red Flag” Approach): Rather than striving for universal imaging access, limited resources should be directed toward establishing rapid referral pathways based on symptom recognition. Training primary care providers to recognize persistent “red flag” symptoms (e.g., pelvic pain, persistent bloating, early satiety) and establishing direct “green channels” to regional tertiary centers can reduce diagnostic delays more effectively than deploying low-quality ultrasound equipment to every village.

### 4.3 Screening-driven thyroid cancer epidemic and global diagnostic disparities

The epidemiological landscape of thyroid cancer has shifted from a pattern driven solely by natural history to one shaped by the complex interplay of surveillance intensity and evolving etiological drivers. While detection bias contributes significantly to the rising incidence, attributing the global surge solely to screening would be an oversimplification. Emerging evidence suggests that the burden of thyroid cancer is driven by a “dual engine”: the expansion of diagnostic scrutiny and a true increase in tumorigenesis linked to modifiable risk factors (42).

First, the contribution of metabolic and environmental factors points to a genuine rise in disease risk. The global surge in obesity and insulin resistance has been identified as a critical driver. *Han et al* (43) confirmed that obesity is not only a risk factor for thyroid cancer but also accounts for the higher prevalence observed in women, likely through hormonal pathways involving estrogen and insulin-like growth factor-1 (IGF-1) which stimulate thyroid cell proliferation. Concurrently, widespread exposure to environmental endocrine-disrupting chemicals (EDCs) and shifts in dietary patterns have altered the biological risk landscape. Increased intake of dietary nitrates—often associated with processed foods and agricultural run-off—has been implicated as a potential thyroid disruptor (44, 45). These factors may explain the observed increase in larger, clinically significant tumors that cannot be attributed to screening artifacts alone.

Second, screening-driven overdiagnosis remains the dominant factor in High-SDI regions. In these settings, the “three-tiered” pattern is characterized by the detection of indolent cases. For instance, a landmark study by Seoul National University highlighted that while the ultrasound detection rate of thyroid nodules skyrocketed from 3% to 67% over two decades, the 10-year progression rate of papillary microcarcinomas remained at only 7%. This discrepancy led to an overtreatment rate reaching 62%, with many patients undergoing unnecessary thyroidectomies (46–48). To mitigate this, High-SDI nations have shifted toward “screening de-escalation.” The American Thyroid Association (ATA) guidelines now link TI-RADS (Thyroid Imaging-Reporting and Data System) grading to stricter fine-needle aspiration (FNA) indications, a policy that has successfully reduced the biopsy rate of low-risk nodules by 35% (49). Furthermore, the integration of AI(Artificial intelligence)-assisted imaging diagnostics and molecular precision screening (e.g., BRAF V600E mutation testing) offers a promising pathway to distinguish high-risk cases from indolent ones, thereby reducing resource waste (50–52).

In contrast, Low-to-Middle SDI countries face a “hidden burden” driven by nutritional imbalances and resource deficits. In iodine deficiency disorder (IDD)-endemic regions, such as parts of South Asia, the etiological profile differs significantly. Follicular carcinoma, a more aggressive subtype with lower sensitivity to ultrasound screening, accounts for up to 38% of cases in these regions, compared to only 8% in High-SDI countries (53, 54). Compounded by the lack of high-frequency ultrasound probes and pathological diagnostic capabilities, more than 65% of patients in these settings are diagnosed at an advanced stage. This paradoxical pattern of “low incidence but high mortality” underscores that for Low-SDI nations, the priority must be balancing iodine supplementation programs to correct nutritional drivers while investing in fundamental diagnostic infrastructure, rather than blindly replicating the intensive screening models of high-income nations.

### 4.4 EAPC trends reveal evolving risk patterns and widening global inequities

EAPC metrics offer a quantitative framework for evaluating the effectiveness of public health interventions. In high-SDI countries, mortality rates and DALYs associated with ovarian and thyroid cancer have shown consistent declines, reflecting the synergistic effects of early screening, health education, and targeted therapies. Conversely, positive EAPC values for multiple cancer types in low-SDI countries indicate either insufficient interventions or compounded risk exposures. These disparities are rooted in generational shifts in risk structures. While traditional behavioral risks such as smoking and alcohol consumption have declined in high-SDI regions, high sugar intake and metabolic abnormalities have become increasingly prominent risk factors (55, 56). In contrast, low-SDI regions continue to face persistent traditional risks that are further exacerbated by occupational and environmental exposures (57). Predictive models suggest that disparities may widen by 2050: high-SDI countries are likely to sustain downward trends in cancer mortality, whereas middle- and low-SDI countries may experience a substantial rise in DALYs related to ovarian and thyroid cancers if timely and targeted interventions are not implemented. This growing divergence underscores the urgent need for globally coordinated strategies to address the shifting landscape of cancer risk and burden.

## 5 Conclusion

In summary, this study provides the first comprehensive description of the incidence trends of three major endocrine-related cancers among reproductive-age women in 15 countries across the Asia-Pacific region. Our findings reveal a polarized landscape exacerbated by a transition from behavioral to metabolic risk drivers: while High-SDI nations face challenges associated with overdiagnosis and Westernized lifestyle risks, Low-SDI nations confront a “survival crisis” stemming from diagnostic delays and resource deficits. To address these widening inequities, we propose a region-specific stratified framework. For High-SDI nations, the focus must shift toward “precision optimization,” specifically targeting metabolic interventions and the de-escalation of thyroid cancer surveillance (e.g., stricter TI-RADS adherence) to curb overdiagnosis. Conversely, Low-to-Middle SDI nations should prioritize “resource-adapted action,” entailing the establishment of symptom-triggered referral pathways, the promotion of opportunistic salpingectomy during obstetric procedures as a primary prevention tool, and the strengthening of basic pathology infrastructure. These tailored strategies, underpinned by international collaboration, are essential to reversing regional disparities and improving health outcomes for this vulnerable population.

## Data Availability

Publicly available datasets were analyzed in this study. This data can be found here: The data used for these analyses are all publicly available at https://ghdx.healthdata.org/gbd-2021.
