# Supplementary figures and images for: Spatial and temporal trend analysis of the burden of endocrine-related cancers among women of reproductive age in the Asia-Pacific region from 1990 to 2021: results based on the GBD study
Source: Front Oncol. 2026 Jan 21;15:1678501. doi: 10.3389/fonc.2025.1678501 (PMC12871539; doi:10.3389/fonc.2025.1678501)

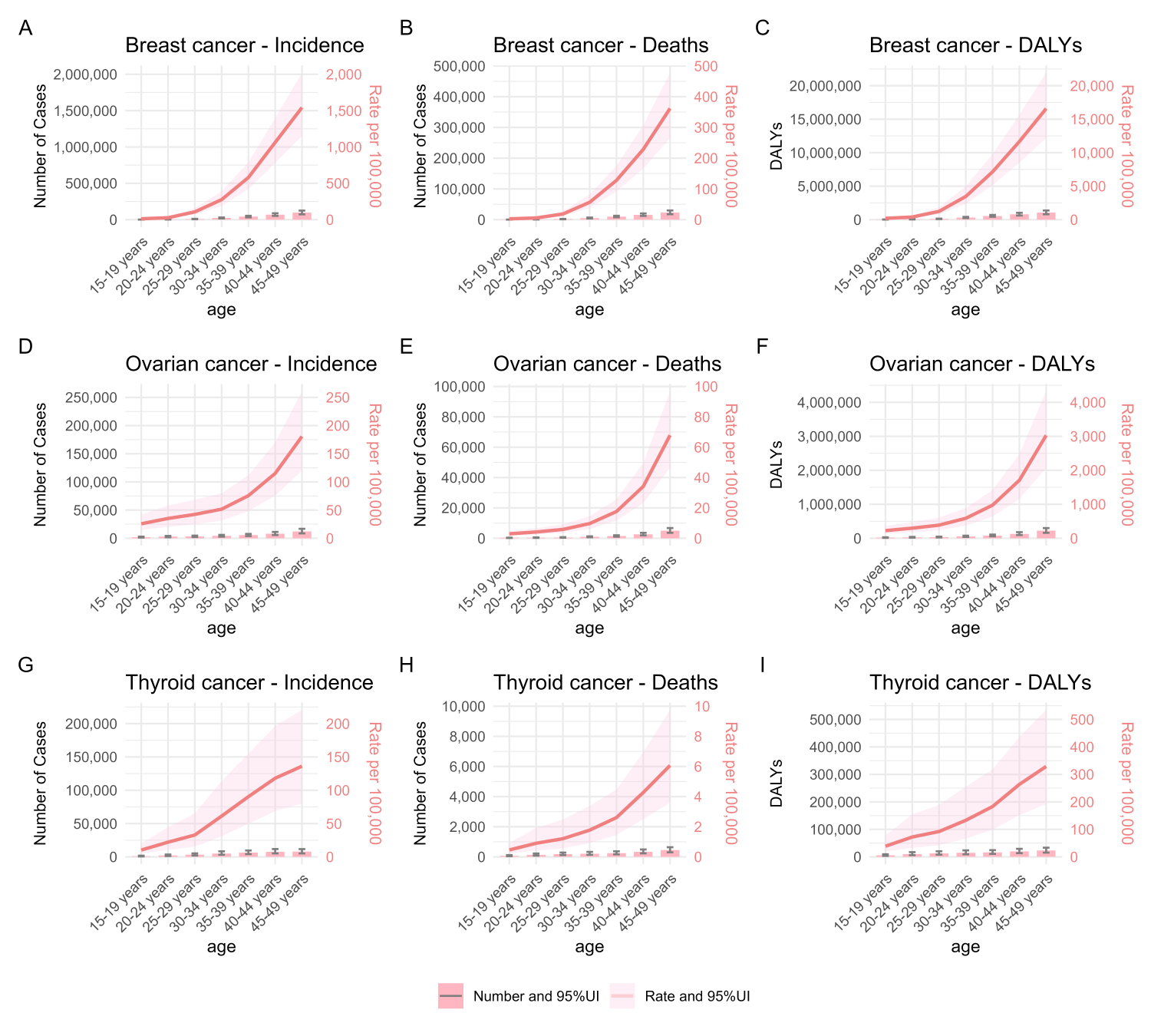

Supplement: Supplementary file 1 [file Image1.tiff]
